# Influence of overweight and obesity on perinatal outcomes in assisted reproduction: a retrospective cohort study

**DOI:** 10.1186/s12884-022-04920-8

**Published:** 2022-07-28

**Authors:** Victoria Campos Dornelles, Marta Ribeiro Hentschke, Mariangela Badalotti, Isadora Badalotti-Teloken, Vanessa Devens Trindade, Bibiana Cunegatto, Natália Fontoura de Vasconcelos, Alvaro Petracco, Bartira Ercília Pinheiro da Costa, Alexandre Vontobel Padoin

**Affiliations:** 1 Fertilitat - Reproductive Medicine Center, Rua Gomes Jardim, 201 Torre Norte 15° andar, Santana, Porto Alegre, Rio Grande do Sul 91530-001 Brazil; 2 School of Medicine, Pontifical Catholic University of Rio Grande do Sul, PUCRS, Av. Ipiranga, 6681, prédio 12A, Partenon, Porto Alegre, Rio Grande do Sul 90619-900 Brazil

**Keywords:** Overweight, Obesity, Infertility, In vitro fertilization, Newborn

## Abstract

**Background:**

In spontaneous pregnancies, maternal weight and gestational diabetes are independent risk factors for macrosomia and large-for-gestational-age newborns. Furthermore, maternal body mass index (BMI) of ≥25 kg/m^2^ is associated with worse neonatal vitality, classified as an Apgar score of < 7 at the fifth minute of life. However, few studies have evaluated the influence of BMI on perinatal outcomes in pregnancies resulting from assisted reproduction. Therefore, this study aimed to analyze whether the perinatal outcomes of assisted reproduction are influenced by BMI.

**Methods:**

This was a retrospective cohort study performed at a reproductive medicine center. Patients undergoing assisted reproduction (2013–2020) were divided into three groups according to their BMI (kg/m^2^): group 1, < 25; group 2, 25–29.9, and group 3, ≥30. In total, 1753 in vitro fertilization embryo transfer cycles were analyzed. Data were expressed as mean ± standard deviation or frequency (%). The analysis of variance and chi-square test were performed for comparison. To determine the participants and number of cycles for these analyses, generalized estimating equations were used, considering *p* < 0.05.

**Results:**

In groups 1, 2, and 3, the rates of live birth were 33.5, 32.3, and 29.9% (*p* = 0.668); preeclampsia were 2.9, 6.1, and 6.3% (*p* = 0.268); small-for-gestational-age newborns were 23, 23.2, and 21.7% (*p* = 0.965); macrosomia were 1.9, 0.9, and 2.7% (*p* = 0.708); Apgar score > 7 at the fifth minute were 97.6, 98.2, and 100% (*p* = 0.616); and preterm birth were 29.6, 30.1, and 35.1% (*p* = 0.970), respectively.

**Conclusions:**

In conclusion, although the three groups had similar perinatal outcomes in this study, the study population was too small for conclusive results. The higher the BMI, the lower the chances of clinically relevant LBR and the higher the chances of premature labor and preeclampsia.

## Background

### Obesity and infertility

One-third of the world population is overweight or obese, which are defined as body mass index (BMI) (kg/m^2^) 25–30 and ≥ 30, respectively, and this prevalence is increasing [1, 2]. In addition to obesity, infertility, defined as the absence of pregnancy after 1 year of unprotected intercourse, is a growing worldwide public health condition, affecting one in four couples in developing countries [3, 4] overweight and obesity are well-known risk factors for female infertility and one of the most important preventable risk factors for negative perinatal outcomes [5, 6]. However, the effect of excess weight on assisted reproduction is still controversial and inconclusive [7].

### Effect of BMI on perinatal outcomes

In spontaneous pregnancies, maternal weight gain is related to a higher than average risk of gestational maternal diseases, such as preeclampsia and gestational diabetes, and to higher than average rates of early miscarriage and cesarean delivery [8]. Consistent evidence shows that maternal weight and gestational diabetes are independent risk factors for macrosomia and large-for-gestational-age (LGA) newborns. Therefore, concerns are growing regarding the birth of newborns in these clinical conditions, which are strongly associated with an increased risk of childhood obesity [9–11]. Czarnobat et al. showed that BMI ≥ 25 kg/m^2^ before pregnancy, excessive maternal weight gain during pregnancy, and maternal diabetes are the most prevalent risk factors for macrosomia and LGA newborns in Brazil [12]. Furthermore, maternal BMI of ≥25 kg/m^2^ is associated with worse neonatal vitality, classified as an Apgar score of < 7 at the fifth minute of life [11].

However, few studies have evaluated the influence of BMI on perinatal outcomes in pregnancies resulting from assisted reproductive techniques (ARTs), and current data are controversial. Kawwas et al. analyzed some perinatal outcomes following ARTs and concluded that pregnancy weight has obstetric implications, with obesity having a negative effect independent of weight gain during pregnancy [13]. Moreover, a recent cohort study performed in China revealed an association between pregnancy weight excess and preterm birth, macrosomia, and LGA newborns in pregnancies after ARTs [14].

### Study’s aim and hypothesis

The present study aimed to analyze whether the perinatal outcomes of ART are influenced by the BMI, with the initial hypothesis that perinatal outcomes are worse with high maternal BMI in pregnancies resulting from ARTs.

## Methods

A retrospective cohort study was performed in a reproductive medicine center located in the south of Brazil, which is considered to be one of the five major reproductive centers of the country and receives patients from the south and southeast Brazil, with a rate of approximately a thousand cycles per year.

Data were collected from the clinic’s electronic medical records from 2013 to 2020. Data regarding perinatal outcomes were recorded in the electronic database according to the patient’s reports after delivery. STROBE (Strengthening the Reporting of Observational Studies in Epidemiology) guidelines for observational studies were followed as guidance in the elaboration of this manuscript [15].

All methods performed in this study were in accordance with the Declaration of Helsinki guidelines and regulations.

### Study population

In the study, 4509 ART cycles from patients undergoing in vitro fertilization (IVF) in the reproductive medicine center from 2013 to 2020 were initially included. After excluding cycles that did not involve fresh embryo transfer (ET) and applying the other exclusion criteria, 1753 ART cycles were finally included in the analysis. Among these cycles, 578 resulted in a live birth, and this sample was divided into three groups according to the women’s BMI: group 1: BMI ≤ 24.9 kg/m^2^, group 2: BMI 25–29.9 kg/m^2^, and group 3: BMI ≥ 30 kg/m^2^. Only cycles resulting in a live birth were considered for statistical perinatal analyses.

The present study population included patients undergoing reproductive treatment at the reproductive medicine center who were mostly Caucasians, had a complete high school education, had a mean age of 37 years, and were economically capable of bearing the ART’s costs. Importantly, this study considered ART cycles and not patients because every new ART cycle performed is considered a new patient when studying the outcomes of ARTs. Some patients underwent more than one ART cycle, and this was considered during statistical analyses performed in this study.

**Fig. 1:**
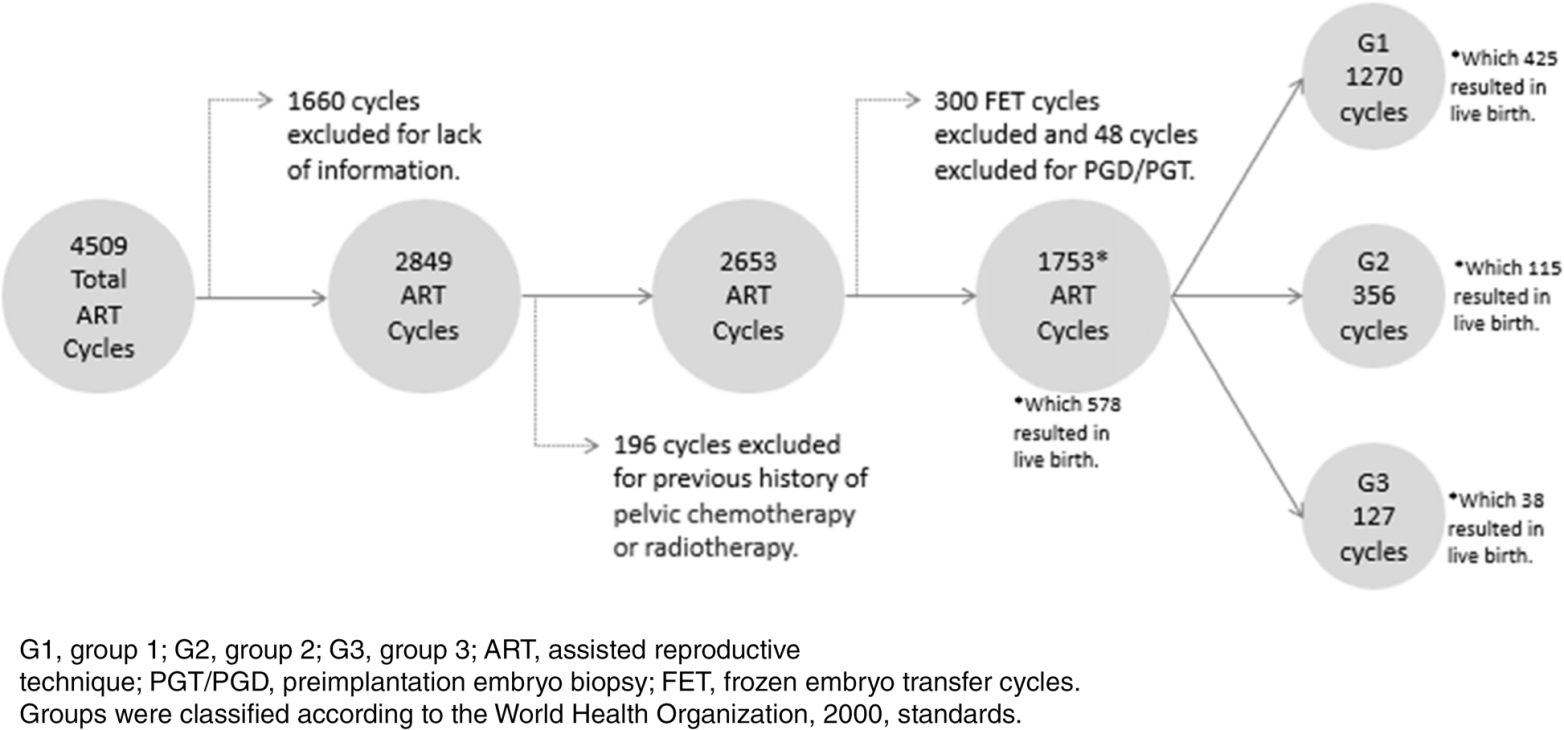
Flowchart of study participants

The study population is presented in Fig. 1.

### Exclusion criteria

Patients with a history of pelvic chemotherapy or radiotherapy and those > 40 years old were excluded because these factors can decrease ovarian reserve and affect maternal outcomes. Furthermore, patients whose embryos were biopsied before implantation were excluded to avoid bias because these embryos are associated with better outcomes in the literature [16].

G1, group 1; G2, group 2; G3, group 3; ART, assisted reproductive technique; PGT/PGD, preimplantation embryo biopsy; FET, frozen embryo transfer cycles.

Groups were classified according to the World Health Organization, 2000, standards.

### Definitions

The neonatal measures considered, such as the Apgar score and weight of the newborn, were according to the pediatrician’s first physical examination performed in the delivery room and registered in patient files. The neonatal percentile was calculated based on World Health Organization standards, which are the basis for Brazil’s Health Minister guides [17, 18]. The newborn classification considering only the birth weight and Apgar index score cutoff for good neonatal vitality were defined according to Brazil’s Health Minister guide, 2011 [18].

### Controlled ovarian stimulation protocol and ET

A controlled ovarian stimulation was performed with gonadotropin-releasing hormone (GnRH) agonist or antagonist protocols according to individual clinical indications, and gonadotropin (75–300 IU/daily) was selected for each patient according to clinical indications. When three or more follicles reached 17 mm or one follicle reached 20 mm, 250 mcg recombinant human chorionic gonadotropin (hCG) or 0.2 mg triptorelin (GnRH agonist) was administered as trigger 34–36 h before ultrasonography-guided oocyte retrieval according to individualized indications. Patients used 600–800 mg of intravaginal micronized progesterone per day. Intracytoplasmic sperm injection was the technique used for all fertilizations in the laboratory, and ETs were performed at the cleavage or blastocyst stage.

The embryo was transferred fresh, hCG in blood samples was analyzed after 10–12 days of ET, and obstetric ultrasound was obtained after 2 weeks of ET to reach a pregnancy diagnosis. When pregnancy was confirmed, prenatal care was provided by the obstetrician chosen by the patient. After delivery, perinatal data were recorded in the clinic’s database according to the patient’s reports.

### Statistical analysis

Statistical analysis was performed using Social Package for Social Sciences (SPSS) for Windows, version 22.0 (SPSS Inc., Chicago, IL, USA), and data were expressed as mean ± standard deviation or frequency (%). Continuous variables were compared using the analysis of variance, and categorical variables were compared using a chi-square test. To determine the patients and number of cycles for these analyses, generalized estimating equations were used considering the difference between the patients and cycles. Statistical significance was defined as *p* < 0.05.

Power calculation was performed using Health Power and Sample Size for Health Researchers, [19] considering 5% alfa, with 1753 cycles obtaining 12% power in the difference for live birth rates (LBRs).

### Ethics

This study was approved by the Research Ethics Committee of the Pontifical Catholic University of Rio Grande do Sul in June 2020 (Protocol 4.085.223). The informed consent from participants was not required because of its retrospective design; nevertheless, all authors signed a data compromise and confidentiality term for collecting data.

## Results

In the whole sample, patients were only subjected to fresh ETs after IVF procedures, and 13% of the patients included were subjected to more than one ART cycle. From this, 19.8% were in G1, 19.8% were in G2, and 21.4% were in G3 (*p* = 0.907). From a total of 578 live births in the sample, 18% resulted from patients subjected to more than one ART cycle, and comparing G1, G2, and G3, the percentage of patients subjected to multiple ARTs were 68, 25.7, and 6%, respectively, *p* = 0.000 (chi-square test was applied in the analysis of these cycles).

The study participants were homogenous, with the three groups having the same percentage of white Caucasian women, high socioeconomic status women, and nonsmokers (*p* > 0.05). Considering this homogeneity, these characteristics were not compared in the group analysis.

The mean maternal age of the total sample was 35.5 ± 3.6 years, and the mean maternal ages of groups 1, 2, and 3 were 35.5 ± 3.6, 35.9 ± 3.6, and 35 ± 4.3 (*p* = 0.040), respectively. The proportion of cycles in young patients (< 25 years) was lower than that in older patients (> 35 years), with younger women in the sample being associated with 100% single cycles (*p* = 0.000, chi-square test). Maternal age adjustment was made for significant differences between the groups.

The mean BMI (kg/m^2^) of the total sample was 23.6 ± 3.7, and the mean BMI values of groups 1, 2, and 3 were 21.7 ± 1.7, 26.8 ± 1.3, and 32.9 ± 2.3 (*p* < 0.05), respectively.

**Fig. 2:**
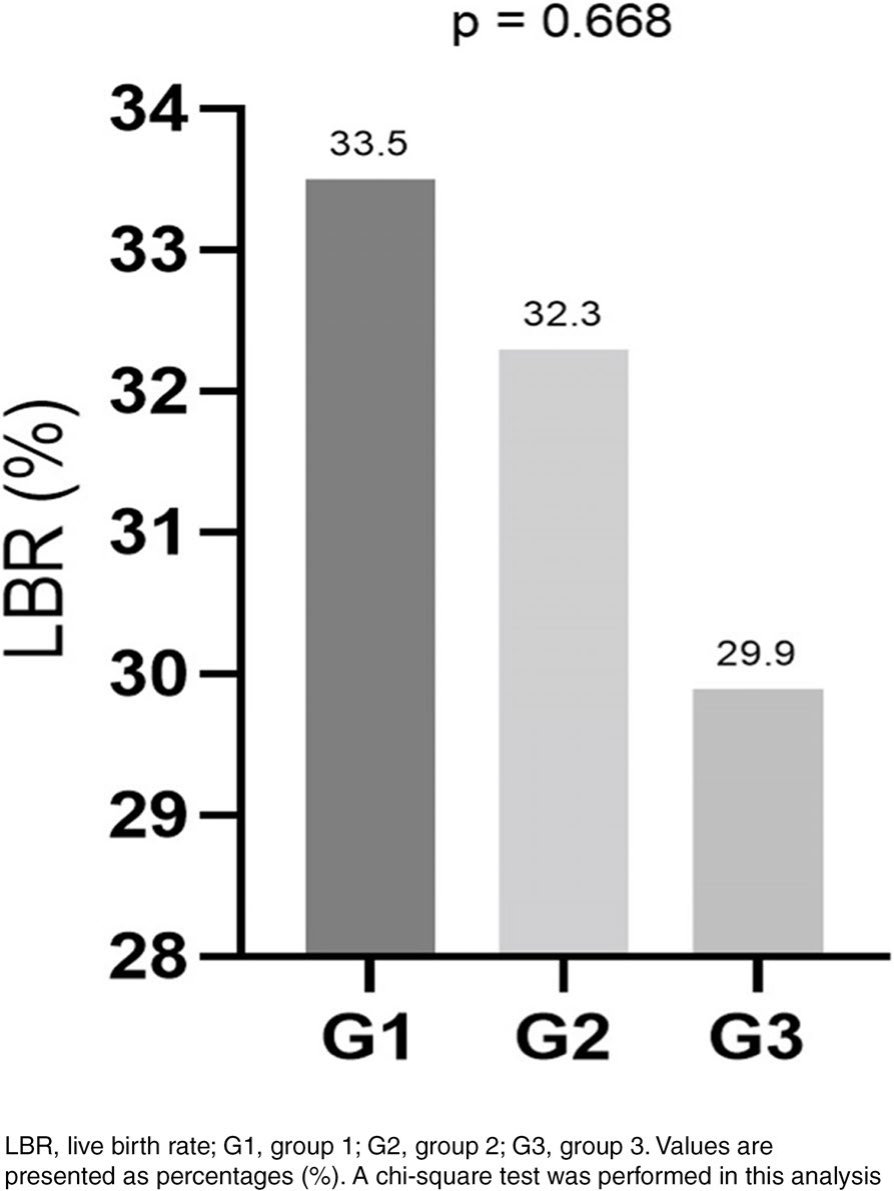
Live birth rate comparison between the groups

Maternal and perinatal outcome analyses are presented in Table 1. Figure 2 illustrates the LBR analysis.

**Table 1:** Perinatal outcomes compared between the groups

Variables	G1 *n* = 1270 cycles *n* = 425 LB	G2 *n* = 356 cycles *n* = 115 LB	G3 *n* = 127 cycles *n* = 38 LB	p
Live birth rate (%)	33.5 CI 20–30	32.3 CI 20–30	29.9 CI 20–30	0.668^b^
Maternal conditions (%)				
Preeclampsia	2.9 CI 2–5	6.1 CI 3–13	6.3 CI 2–22	0.268^a^
PROM	1.3 CI 1–3	4 CI 1–9	3.1 CI 0–19	0.235^a^
Hypothyroidism	15.4 CI 3–8	7.6 CI 1–8	11.1 CI 0–19	0.449^a^
Pregnancy (%)				
Twins	25.2 CI 20–30	26.5 CI 20–30	27 CI 20–30	0.940^a^
Delivery (%)				
C-section	91.4	95.6	97.3	0.221^a^
Vaginal	8.6	4.4	2.7	0.221^a^
Newborn’s (%)				
Percentile				
AGA	72.8 CI 70–80	73.9 CI 64–84	73.9 CI 55–88	0.999^a^
SGA	23 CI 15–25	23.2 CI 13–32	21.7 CI 8–39	0.965^a^
LGA	4.2 CI 2–7	2.9 CI 1–11	4.3 CI 1–26	0.890^a^
Birth weight				
Normal	71.6 CI 70–83	72.6 CI 72–87	78.4 CI 71–95	0.693^a^
ELBW	2.1	0.9	0	0.693^a^
VLBW	4.0	3.5	0	0.693^a^
LBW	20.5	22.1	18.9	0.693^a^
TL	26.6 CI 20–30	26.5 CI 20–30	18.9 CI 18–20	0.579^a^
MS	1.9 CI 1–2	0.9 CI 1–2	2.7 CI 2–3	0.708^a^
Apgar score at the fifth minute				
< 7	2.4	1.8	0	0.616^b^
> 7	97.6 CI 90–99	98.2 CI 93–99	100 CI 82–100	0.616^b^
Outcomes				
CMF	1.4	1.8	0	0.725^b^
PTB	29.6	30.1	35.1	0.970^a^
PND	1.6	1.8	0	0.728^b^
ICU	7.2	2.7	2.7	0.166^a^

G1, group 1; G2, group 2; G3, group 3; *LB* Live birth, *CI* 95% confidence interval, *PROM* Premature rupture of membranes, *AGA* Appropriate for gestational age, *SGA* Small for gestational age, *LGA* Large for gestational age, *ELBW* Extremely low birth weight, *VLBW* Very low birth weight, *LBW* Low birth weight, *TL* Total birth weight less than normal, *MS* Macrosomia, *CMF* Congenital malformations, *PTB* Preterm birth, *PND* Postnatal death, *ICU* Intensive care unit

Values are presented as percentages (%)

^a^Generalized estimating equations

^b^Chi-square test/post hoc

## Discussion

The objective of the present study was to analyze whether the perinatal outcomes of ARTs are affected by maternal BMI. The results suggest no differences in perinatal outcomes based on BMI, but we cannot conclude that the BMI has no effect on perinatal outcomes because the study population was too small to provide conclusive results, and BMI appears to negatively affect perinatal outcomes.

### Perinatal ART outcomes

In this study, the LBR showed clinically relevant worsening of maternal health with obesity, although nonsignificantly. Furthermore, this low LBR pattern was observed by Moragianni et al. who reported that the LBR decreased with an increase in maternal weight [20]. Similarly, Kawwas et al. reported that the LBR decreased with an increase in the BMI (approximately 38.6% in eutrophic BMI, reducing to 29.4% in obese patients), as well as pregnancy rates decreased (approximately 46.1% in eutrophic BMI, reducing to 38.8% in obese patients) [13]. The loss of only approximately 4% chance of live birth in obese patients in the present study is probably due to this study’s limited sample size and still could be clinically relevant for infertile couples.

The C-section rate in the present study was considered extrapolating the 10% that is worldwide recommended by World Health Organization [21]; however, this result is expected in the population analyzed because Brazil’s C-section rate is > 55% considering all the delivery types in the country, and it has the second highest rate of C-section in the world, particularly due to its archaic culture [22]. Furthermore, according to national statistics data from Brazil, this rate is even higher among people having a high socioeconomic status (approximately 85% of all deliveries). Because this study population included patients subjected to ART and this treatment in Brazil is expensive, the patients in this sample were considered to have an extremely high socioeconomic status and, thus, the C-section rate was according to the country’s realistic expectations.

Regarding maternal conditions, a nonsignificantly higher prevalence of preeclampsia was observed in overweight and obese women than in eutrophic women, and furthermore, this is well demonstrated in spontaneous pregnancies [12]. Sufficient data were lacking for the statistical analysis of gestational diabetes, and the absence of this prevalent condition in our results is due to a lack of sufficient records.

Regarding the weight of newborns, no difference was observed in the neonatal percentile although the proportion of LGA babies was higher in the obese group than in the overweight group. Furthermore, although no statistical difference was observed, the proportion of low birth weight newborns was the lowest in the obese group, which also had the highest proportion of macrosomia. The Apgar score was mostly > 7 in the fifth minute for both the groups, indicating that most babies were born with good vitality. Moreover, the groups had similar proportions of congenital malformations, postnatal death, and intensive care unit (ICU) needs. These results were different from the data of spontaneous pregnancies, which show higher LGA babies and worse Apgar scores in obese pregnant women [11]. Furthermore, Qu et al. demonstrated a higher risk of LGA babies after ART in overweight and obese women than in eutrophic women. Additionally, Qu et al. compared fresh and frozen ET cycles and showed a worsening of the BMI impact on LGA with fresh ET cycles [14].

Considering the birth weight of newborns individually, the total number of babies with low birth weight was nonsignificantly less in obese patients, unlike the results in the study by Kawwas et al., which presented higher proportions of low birth weight babies in underweight and obese groups. Nevertheless, our study showed a nonsignificantly higher proportion of macrosomia in obese women than in eutrophic women; similar results were obtained by Qu P et al. in ART pregnancies and by Czarnobay et al. in spontaneous pregnancies [12, 14].

In this study, preterm newborns tended to be higher than average in overweight and obese groups, but the results were nonsignificant even when groups 2 and 3 together were compared with group 1. However, the high frequency of preterm newborns in overweight and obese groups is consistent with the findings of Kawwas et al. who showed a higher than average prevalence of preterm newborns among obese women. Moreover, Qu found a higher prevalence of preterm newborns in overweight and obese women than in eutrophic women [13, 14].

The proportions of pregnancies with twins were similar among the groups in this study, suggesting that a nonsignificantly high proportion of preterm babies in the obese group was not associated with it. Furthermore, a nonsignificantly higher ICU indication was observed in group 1, which was probably related to newborns’ birth weight, as most of the indications in our sample were related to low birth weight, which was slightly higher than normal in this group of patients. This explains why it was less observed in overweight and obesity. Furthermore, it is important to consider, once again, the major eutrophic sample size in this analysis. No newborn with congenital malformations or postnatal death was observed in the obese group, which may be because of the limited sample size.

### Study’s limitations

As this was a retrospective study, a major limitation was the possibility of incomplete medical records and a cohort with predefined sample size. Furthermore, evaluating women’s weight throughout the pregnancy was not possible. Moreover, some patients may have reported incorrect information regarding pregnancy, labor, and personal characteristics. Thus, another limitation of this study was a lack of detailed population characteristics, with the provision of only general population characteristics.

Additionally, child growth standards were chosen rather than newborn standards because only information for calculating child growth was available, and this was another limitation of this retrospective study because the child growth standards do not account for gestational age at birth.

The present study’s limited sample size with only 12% statistical power could produce conclusive results. We could not conclude that the BMI did not affect perinatal outcomes because the chances of detecting statistical differences between the results were low. Some of our nonsignificant results are still clinically relevant. A larger sample size with 80% power could bring statistical significance to these analyses.

A controlled prospective study is important for further conclusions, with not only the inclusion of other weight measures, such as the abdominal circumference but also the comparison of ARTs with spontaneous pregnancies.

## Conclusions

In conclusion, although perinatal outcomes did not differ according to the BMI in this study, the study population was too small, and hence, we could not confirm that the BMI has no effect. LBR appeared to decrease with an increase in BMI, and the risks of preeclampsia and preterm labor appeared to increase with an increase in BMI; however, the differences were nonsignificant. These findings are clinically relevant for patients seeking pregnancy with ART.

The lack of significant differences in perinatal results between the groups, unlike in other studies, could be explained by the limited sample size and low statistical power. A sample with 80% power can bring significance to some of the tendencies found.

For conclusive results, further studies are needed that will consider the patients’ weight gain during pregnancy and compare pregnancies resulting from ARTs with spontaneous pregnancies. Although our study showed no significant effects of BMI on perinatal outcomes, we recommend the maintenance of healthy weight before pregnancy because consistent evidence has shown worse outcomes of weight excess in spontaneous pregnancies.

## Data Availability

All data generated or analyzed during this study are included in this published article.

## Abbreviations

BMI: Body mass index
ART: Assisted reproductive technique
LBR: Live birth rate
LGA: Large for gestational age
ET: Embryo transfer
IVF: In vitro fertilization
GnRH: Gonadotropin-releasing hormone
hCG: Human chorionic gonadotropin
SPSS: Social Package for Social Sciences
ICU: Intensive care unit
